# Ischemic Retinopathies: Oxidative Stress and Inflammation

**DOI:** 10.1155/2017/3940241

**Published:** 2017-12-19

**Authors:** José Carlos Rivera, Rabah Dabouz, Baraa Noueihed, Samy Omri, Houda Tahiri, Sylvain Chemtob

**Affiliations:** ^1^Department of Ophthalmology, Maisonneuve-Rosemont Hospital Research Center, University of Montréal, Montréal, QC, Canada; ^2^Department of Pediatrics, Ophthalmology and Pharmacology, Centre Hospitalier Universitaire Sainte-Justine Research Center, Montréal, QC, Canada

## Abstract

Ischemic retinopathies (IRs), such as retinopathy of prematurity (ROP), diabetic retinopathy (DR), and (in many cases) age-related macular degeneration (AMD), are ocular disorders characterized by an initial phase of microvascular changes that results in ischemia, followed by a second phase of abnormal neovascularization that may culminate into retinal detachment and blindness. IRs are complex retinal conditions in which several factors play a key role during the development of the different pathological stages of the disease. Increasing evidence reveals that oxidative stress and inflammatory processes are important contributors to the pathogenesis of IRs. Despite the beneficial effects of the photocoagulation and anti-VEGF therapy during neovascularization phase, the need to identify novel targets to prevent initial phases of these ocular pathologies is still needed. In this review, we provide an update on the involvement of oxidative stress and inflammation in the progression of IRs and address some therapeutic interventions by using antioxidants and anti-inflammatory agents.

## 1. Introduction

Ischemic retinopathies (IRs), such as retinopathy of prematurity (ROP), diabetic retinopathy (DR), and age-related macular degeneration (AMD), are the main causes of severe visual impairment and sight loss in children, adults (with diabetes), and elderly population, respectively [1, 2]. IRs are biphasic diseases characterized by loss of the preexisting vessel bed and sustained hypoxia that leads to a secondary vasoproliferative phase characterized by anarchic vessel proliferation into the vitreous humor, which can result in retinal detachment and blindness [3]. Importantly, the ensuing ischemic events secondary to initial vessel loss can also have devastating effects on neuronal homeostasis and function [4].

Several factors contribute to the pathogenesis of IRs; however, oxidative stress [5, 6] and inflammatory processes [7, 8] stand as major ones. Oxidative stress is defined as an imbalance favoring generation over the removal of reactive oxygen species (ROS), such as free radicals, nitric oxide (^•^NO), superoxide anion (O_2_^−•^), and hydrogen peroxide (H_2_O_2_). Free radicals are short-lived reactive molecules that disrupt molecular nature of lipids, amino acids, and nucleic acids. ROS are normal by-products of aerobic metabolism; inefficient removal by antioxidants leads to redox injury and cellular damage [9]. Factors that participate in the pathogenesis of IRs, such as hyperoxia in ROP, hyperglycemia in DR, and lipid accumulation in AMD, are important amplifiers of oxidative stress that cause dysregulation of cell metabolism and participate in limiting antioxidant defenses during the development of the IRs [9–13].

Inflammation and oxidant stress are tightly intertwined. Inflammation is a cellular response to factors (including those due to oxidant stress) that challenge the homeostasis of the tissues, but this process also acts as a defense mechanism to maintain the equilibrium of the functions. Cytokines and chemokines are signaling proteins that travel throughout the body to exert specific functions in inflammation. However, sustained inflammation can be detrimental to tissue integrity. Increasing evidence shows that a local and/or systemic augmentation of ROS or inflammatory molecules is implicated in the pathogenesis of IRs. Current therapies only target late phases of these ocular pathologies, specifically the vasoproliferative phase. Yet, there is an urgency to tackle the initial ischemic phases. We hereby review prominent concepts that involve oxidative stress and inflammation in the genesis and progression of IRs.

## 2. Retinopathy of Prematurity (ROP)

ROP is the major cause of visual impairment and blindness in neonates worldwide. A demographic census in 2010 reported ~184,700 preterm babies worldwide with ROP; 20,000 of them were blind or severely visually impaired [14]. This problem is reaching epidemic proportions in middle-income and developing countries; the survival of extremely premature infants is increasing without a significant change in morbidity [15].

### 2.1. Pathogenesis of ROP

ROP is a disease that affects the immature retinal vascular system and thus occurs in premature infants with an incompletely vascularized retina. Therefore, the incidence and severity of retinopathy are directly proportional to the degree of prematurity. It is widely accepted that the development of ROP progresses through two phases. The first phase begins when retinal vascular growth ceases after premature birth. During this time, the retinal cytoprotective factors, such as insulin-like growth factor-1 (IGF-1), diminish [16] and the vessels become particularly vulnerable to injury caused by any number of stressors, including the amount of oxygen supply. Premature infants are exposed to higher oxygen tension after birth compared to those in utero. This leads to a downregulation of the major hypoxia-driven vascular endothelial growth factor (VEGF), as well as an increase in vasoobliteration of immature retinal capillaries through the actions of oxidant stress and intertwined inflammation [17]. The loss of blood vessels, associated with an increase in maturation-dependent metabolic demand, causes the retina to become gradually hypoxic. In order to ensure an adequate perfusion to the hypoxic retina, an overproduction of hormones and growth factors stimulates an excessive vessel formation at the junction between the vascular and avascular retina. This sets the beginning of the second phase of ROP. Interestingly, these new vessels fail to reperfuse the avascular retina, as instead of growing into areas of need, they grow chaotically into the vitreous, where traction and detachment of the retina as well as bleeding can occur, ultimately resulting in blindness. This critical stage of ROP (defined in humans as stage 4-5) occurs most frequently around 34–36 weeks after conception [18].

### 2.2. Nitrooxidative Stress, Lipid Oxidation and Nitration, and ROP

**Figure 1:**
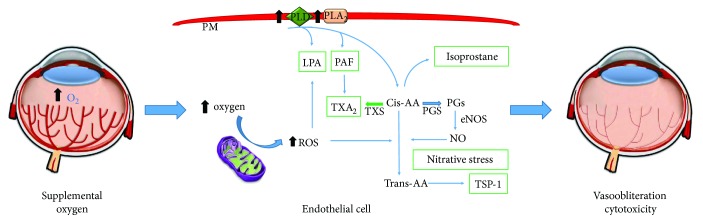
The effects of oxidant stress on premature retinal vasculature. The premature retina is relatively deficient in antioxidants. Consequently, oxidant stress is more likely to induce peroxidation and nitration that is cytotoxic to retinal microvasculature. Downstream mediators of peroxidation, notably the phospholipids PAF and LPA, the nonenzymatically derived prostanoids, isoprostanes, and nitration products such as *trans*-arachidonic acids (TAAs), are all cytotoxic to retinovascular endothelium, causing vasoobliteration. PLD: phospholipase D; PLA_2_: phospholipase A2; PM: plasma membrane; PGS: PG synthase; TXS: thromboxane synthase; TSP-1: thrombospondin-1.

The pathogenesis of ROP is related to many causative factors, including low gestational age, low birth weight, genetic components, and relative hyperoxia. Hyperoxia is one of the major environmental predisposing factor to ROP, as it is the molecular basis for generation of reactive oxygen species (Figure 1). Nitric oxide formation also requires oxygen, and based on the redox state of the retina, it can have either beneficial or detrimental effects to the retina. Using the experimental model of oxygen-induced retinopathy (OIR) which shares many features of ROP, it was shown that endothelial nitric oxide synthase expression and activity increase when the redox state is shifted towards an oxidative environment [19]. Under these conditions, nitric oxide reacts with reactive oxygen species resulting in generation of nitrites, nitrates, and most damaging peroxynitrite that cause retinal microvascular degeneration [20] by a process called nitrooxidative stress. Genetic ablation [21] and pharmacologic inhibition of endothelial nitric oxide synthase [22] have been shown to attenuate hyperoxia-induced retinal microvascular degeneration, demonstrating the importance of nitrooxidative stress in ROP.

Lipid peroxidation of cell membranes secondary to inadequately high oxygen tension is pivotal to the pathogenesis of ROP (Figure 1). The retina is highly susceptible to lipid peroxidation, being composed of lipids with elevated levels of polyunsaturated fatty acids (PUFAs), such as docosahexaenoic acid (DHA), *cis*-arachidonic acid, and choline phosphoglyceride. Prostanoids are synthesised from arachidonic acid by the sequential action of phospholipase A_2_ and cyclooxygenase, which are triggered by oxidant stress and peroxidation. The accumulation of peroxides eventually favours thromboxane A_2_ production, which is a potent cytotoxic agent in microvessels [23]. Inhibitors of cyclooxygenase and thromboxane A_2_ synthase selectively curtail oxygen-induced retinal vasoobliteration in mice [23]. Nitrative stress results in *cis*- to *trans*-isomerisation of arachidonic acid, and this was shown to contribute to retinal vascular degeneration in a mouse model of ROP [24]. Circulating levels of plasma *trans*-arachidonic acid are increased in oxygen-induced microvascular degeneration and are known to be secondary to nitrative stress. More specifically, *trans*-arachidonic acid formation has been shown to be abrogated in mice treated with nitric oxide synthase inhibitors and in mice deficient in endothelial nitric oxide synthase [22]. The endothelial cytotoxicity induced by *trans*-arachidonic acid results from the formation of the antiangiogenic and proapoptotic thrombospondin-1 [22] via activation of long-chain fatty acid receptor GPR40 [25].

Platelet-activating factor and lysophosphatidic acid are other lipids generated during peroxidation that act as proinflammatory mediators and contribute to microvascular injury in the retina. Platelet-activating factor is abundantly generated during oxidant stress, and its cytotoxic effects are mediated to a large extent by thromboxane A_2_ [26]. Along the same lines, lysophosphatidic acid is released from lysophosphatidylcholine by the action of lysophospholipase D and can play a role in retinal inflammation leading to microvascular cytotoxicity in OIR [27].

#### 2.2.1. Antioxidant Agents in ROP

Susceptibility of the immature retina to hyperoxia-triggered oxidative stress lies in the incomplete development of its antioxidant system [28, 29]. In order to circumvent this hurdle, supplementation with antioxidants has been attempted. Vitamin E is a naturally occurring free radical scavenger that decreases lipid peroxidation and helps to maintain membrane integrity in retinal cells. Vitamin E normally occurs in its highest concentration in the retina; however, premature infants are born with approximately 10% of adult levels [30]. A few clinical trials have showed a benefit of vitamin E supplementation on the incidence and severity of ROP [31, 32], but these effects have mostly been marginal [33]. In addition, adverse effects from vitamin E supplementation have been reported, resulting in an increased risk of life-threatening infections and bleeding in the brain when injected intravenously [34]. Hence, prophylactic supplementation with vitamin E remains controversial and is currently not regularly utilized.

Vitamin C is another important aqueous phase antioxidant in cells and plasma [35]. Vitamin C has a number of important metabolic functions and is actively transported across the placenta [36]. Vitamin C concentrations in cord plasma are higher than the mother's and, in term infants, plasma concentrations fall considerably over the first 24 hours of life [37]. Preterm infants generally have higher cord vitamin C concentrations than term infants, and concentrations then decline over a few days [38]. Most preterm infants receive vitamin C as part of a multivitamin supplement, but there are few data on which to base optimum concentrations [39]. In fact, the relation between vitamin C concentration and morbidity in very preterm infants remains controversial. Silvers et al. [40] reported that high plasma vitamin C concentrations were associated with a low antioxidant status and poor outcome in premature infants, as well as, a greater risk of developing bronchopulmonary dysplasia [41]. In contrast, Moison et al. [42] reported lower plasma vitamin C concentrations in preterm infants who developed bronchopulmonary dysplasia compared with those who did not. In a pilot observational study in very low birth weight infants, an increased risk of ROP with higher plasma vitamin C concentrations at day 7 and an increased risk of bronchopulmonary dysplasia with lower concentrations at 28 days were found [43]. Darlow et al. [44] thus hypothesized that maintaining a lower plasma vitamin C concentration in the first week of life and a higher concentration in weeks 3-4 would be accompanied by improved clinical outcome and least morbidity (chronic lung disease and ROP) in very low birth weight infants.

### 2.3. Inflammation and ROP

The role of inflammation in ROP has been poorly investigated. Recent evidences suggested that prenatal, perinatal, and postnatal inflammation might contribute to a gradual increase in the risk for ROP [7]. Clinical studies found that inflammatory stimuli such as bacteria in the placenta [45] and late bacteremia [46] were risk factors for developing ROP. Moreover, systemic inflammation in animal models in neonates has been shown to perturb retinal vessel development and to induce pathological features of ROP [47, 48]. Furthermore, recent studies by using genetically modified mice with a deficiency in tetrahydrobiopterin (BH4), an essential cofactor implicated in multiple metabolic process, showed that BH4 plays an essential role in maintaining the inflammatory and neurovascular retinal homeostasis [49] and is involved in the development of retinopathy [50].

Cytokines and chemokines are small proteins secreted by immune cells that play a central role in distinct inflammatory processes including the progression of ROP [51]. Cytokines such as IL-1*β*, TNF*α*, and IL-6 act as primary initiators of inflammation following infection or tissue damage [52]. Interestingly, IL-1*β* and TNF*α* produced by retinal microglia cells following exposure to hypoxia have been associated with deleterious effects in the retina [53]. In OIR model, IL-1*β* has been associated with retinal microvascular degeneration by inducing semaphorin3A in neurons [54], while in the choroid IL-1*β* causes direct cytotoxicity to choroidal blood vessels, which results in a hypoxic subretina and consequently loss of retinal pigment epithelium (RPE) and photoreceptor integrity [55].

IL-10 is generally considered an anti-inflammatory cytokine [56], capable of protecting the developing retina against ongoing inflammation. Although a study showed that IL-10 can be implicated in promoting pathological angiogenesis in an OIR mice model [57], in another study, IL-10 was able to inhibit the expression of proinflammatory cytokines on microglial cells [58]. Furthermore, in pregnant rats exposed to systemic inflammation, IL-10 treatment reduced the occurrence of brain damage in their newborn pups [59]. Infants with an IL-10 high-producer allele showed a trend (albeit not significant) towards a lower prevalence of severe ROP [60].

On the other hand, chemokines which induce chemotaxis and regulate movements of immune cells such as microglia to sites of inflammation are of special interest for pathophysiology of ROP. For instance, the chemokine interleukin-8 (IL-8) is implicated in both inflammation and pathological neovascularization in the eye [61]. In humans, higher serum concentration of IL-8 right after birth was associated with later ROP [62]; concordantly in rats, increased levels of an IL-8 homologue were detected during the peak of pathological neovascularization in a model of ROP [63].

An important player in innate immunity called RANTES is suggestive of participating in the development of ROP. Although the role of RANTES in ROP is not known, low concentrations of RANTES have been detected in the vitreous humor of patients with vasoproliferative ROP [64]; low serum levels have also been detected in infants who later developed severe ROP [65, 66]. Further investigations are needed to establish a more convincing role for RANTES in ROP.

MCP-1 is an attracting factor in a variety of immune cells and is expressed in a wide range of tissues including activated microglia in the neuroretina [67]. Preterm infants who later developed ROP tended to have higher cord serum concentrations of MCP-1 than healthy preterm infants [68]. Elevated levels of MCP-1 in the vitreous humor of patients with retinopathy have been documented [69, 70]. In animal models, MCP-1 was associated with retinal neovascularization, possibly by attracting macrophages/microglia during the ischemic phase of retinopathy [71, 72].

#### 2.3.1. Anti-Inflammatory Agents in ROP

Few pharmacological interventions using anti-inflammatory drugs have been tried in ROP. Ketorolac is a nonsteroid anti-inflammatory drug (NSAID) derived from indomethacin that inhibits the synthesis of prostaglandins by competitively blocking the activity of COX 1 and COX 2 [73]. Upon topical application, ketorolac diminishes prostaglandin E_2_ concentration in aqueous humor [74]. A recent preliminary report suggests that ketorolac in the form of an ophthalmic solution can reduce the risk of developing severe ROP in very preterm newborns, without producing significant adverse side effects [75]. Interestingly, the authors showed that the incidence of severe ROP was significantly lower in very preterm newborns treated with ketorolac, when compared to the controls not receiving such treatment. These results suggest that administration of ketorolac as an ophthalmic solution might be an effective preventive strategy in patient at risk of developing severe ROP.

Dexamethasone, a steroidal anti-inflammatory agent, has also shown to reduce the incidence of ROP [76]. However, its use is associated with significant side effects [77, 78], and its efficacy in preventing ROP is controversial. A prospective randomized, controlled multicenter clinical trial to investigate the benefits of COX inhibitors for prevention of ROP is proposed (NCT02344225).

An anti-IL-1 treatment using a proprietary drug candidate labelled 101.10 (amino acid sequence: rytvela) has shown promising results in animal models. Rivera et al. demonstrated pronounced beneficial effects of 101.10 in a rodent model of ROP [54]. Interestingly, 101.10 markedly reduced retinal inflammation and preserved retinovascular architecture in the animals exposed to the OIR model. However, human clinical trials are still needed to investigate the benefits of 101.10 in preventing ROP progression.

#### 2.3.2. Promising Therapeutic Treatments in ROP

The Caffeine for Apnea of Prematurity (CAP) trial found that caffeine was beneficial in reducing the incidence of severe ROP [79]. Mechanisms implicated in caffeine actions in reducing ROP are still unknown, yet effects on sonic hedgehog [80], matrix metalloproteinases (MMPs) [81], and oxidative stress [82] could be involved. Up to now, a prospective randomized, controlled multicenter clinical trial to investigate the benefits of caffeine on ROP is ongoing (NCT02344225).

Beneficial properties of omega-3 supplementation in ROP have been suggested. Connor et al. [83] showed that dietary omega-3 fatty acids protect against pathologic neovascularization by increasing the formation of cytoprotective and anti-inflammatory metabolites. A meta-analysis showed that long-chain polyunsaturated fatty acid supplementation of infant formulas improves infants' visual acuity up to 12 months of age [84]. The impact of omega-3-PUFA supplementation specifically on human ROP has started to be addressed; omega-3-containing fish oil emulsion supplementation to premature infants has been shown to be associated with reduced risk for ROP [85]. At present, a trial examining the effects of omega-3 PUFA supplementation to very low birth weight infants on prevention of ROP is ongoing at the University of California (NCT02486042).

## 3. Diabetic Retinopathy (DR)

DR is a leading cause of vision loss in working adult population and one of the most common complications of diabetes mellitus [1, 86]. It is estimated that the prevalence of diabetes mellitus in adults (aged 20–79 years) will continue to rise in the following years [87]. Therefore, as a result of urbanization and aging population, the number of DR patients is projected to increase from 37.3 million to 56.3 million by 2030 [88].

### 3.1. Pathogenesis of DR

**Figure 2:**
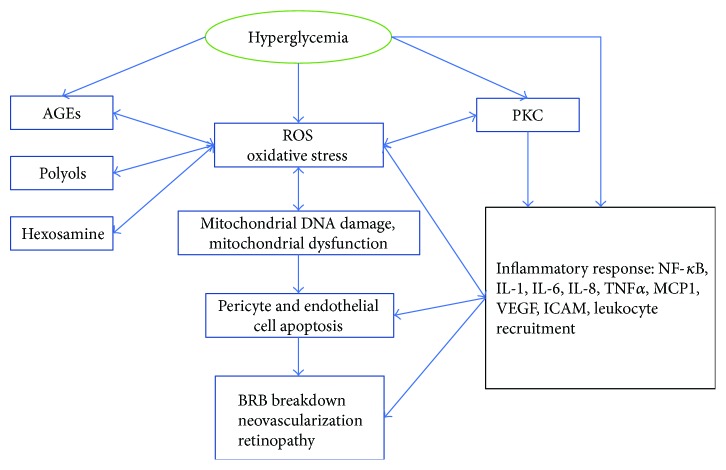
The role of oxidative stress and inflammation in diabetic retinopathy. Hyperglycemia activates PKC, AGEs, polyol, and hexosamine pathways which contribute to oxidative stress and mitochondrial dysfunction leading to pericyte and endothelial cell apoptosis. Upregulation of inflammatory mediators results in cell death and BRB breakdown.

DR is a progressive disease that develops in stages, from mild nonproliferative DR to moderate severe nonproliferative DR and finally to the ultimate stage of proliferative DR, which is characterized by the growth of abnormal leaky retinal blood vessels and consequently to the detachment of the retina [89]. Throughout the different stages, patients with DR may develop diabetic macular edema (DME) which is due to the breakdown of the blood-retinal barrier (BRB) leading to a vascular leakage of fluid and plasma components at the retina [90]. The inner BRB consists of a single layer of tightly connected endothelial cells which is supported by pericytes. A preserved BRB plays a key role in supporting and maintaining the integrity of the retina and prevents the retinal vessels from leaking [91, 92]. Different factors have been shown to contribute to the BRB breakdown (Figure 2). During diabetes, high glucose levels cause an impairment of the tight junctions which become loosened, and endothelial cells and pericytes undergo apoptosis and thus allow an outward flow of plasma components, including lipid and proteins, into the vitreous. BRB leakage results in the swelling of the macula associated with improper perfusion and development of areas of retinal ischemia [92, 93]. In addition, circulating leukocytes, which become less deformable, adhere with the activated endothelial and participate in capillary occlusion and ischemia [94].

Several studies have shown that vascular endothelial growth factor (VEGF) has a primordial role in the BRB breakdown. VEGF levels are increased in patients with proliferative DR and contribute to retinal vascular permeability [95]. Moreover, VEGF upregulation occurs even before the onset of hypoxia. Indeed, it has been shown that VEGF levels are increased at the early stages of DR, and this could be a consequence of an inflammatory environment characterized by the release of proinflammatory cytokines (i.e., IL-1*β* and IL-6) and the formation of advanced glycosylation end products (AGEs) [96, 97]. In response to hypoxia and inflammation, VEGF in association with angiopoietin 2 (Ang2) plays a key role in neovascularization and affects the integrity of preexisting vasculature [98]. VEGF antagonists attenuate vascular leakage in DR [99].

Pericyte recruitment to the microvessel wall is primordial for the formation of BRB, and this recruitment is controlled by the platelet-derived growth factor (PDGF) B and PDGF receptor *β* (PDGFR*β*) [100]. Human and animal studies have shown that the loss of pericytes in diabetes is triggered by the activation of nuclear factor kappa B (NF-*κ*B) with an increase in Bax expression causing pericyte apoptosis [101]. Another study has shown that high levels of glucose activated protein kinase C*δ* (PKC*δ*) and p38*α* mitogen-activated protein kinases (MAPK) which increase the expression of Src homology 2 domain-containing phosphatase (SHP-1), a protein tyrosine phosphatase, resulting in the dephosphorylation of PDGFR*β* to induce pericyte apoptosis and acellular capillaries in an NF-*κ*B-independent pathway [102]. Pericyte loss is one of the main characteristics of DR with the formation of microaneurysms and acellular capillaries [103].

A new hypothesis suggests that photoreceptors in the outer retina might also play an important role in the development of diabetic retinopathy [104]. Du and collaborators have proposed that photoreceptors contribute to diabetes-induced degeneration of retinal capillaries [105]. Accordingly, diabetes causes oxidative stress in photoreceptors in part through alteration in ion flux. These abnormalities might affect intermediate cells such as Müller cells and leukocytes which result in characteristic pathologic alteration to the retinal vasculature including increased permeability and nonperfusion [104]. In support to this hypothesis, it was demonstrated that DR was less severe in a group of patients with retinitis pigmentosa [106] and in mice lacking photoreceptors [107].

### 3.2. Oxidative Stress and DR

The retina is rich in polyunsaturated fatty acids and characterized by a high-energy demand and an exposure to light; together, these conditions favor oxidative stress. Oxidative stress is involved in the pathogenesis of DR, and high levels of ROS have been found in patients with DR [108].

Mitochondria are the major source of ROS or reactive nitrogen species (e.g., superoxide and peroxynitrite). In presence of high glucose, oxidation of carbohydrates leads to an impairment of the electron transport chain and results in the accumulation of electrons at coenzyme Q leading to the generation of superoxide anion from oxygen, which in turn generates other ROS [109]. ROS play a role in the production of cytokines to promote inflammation and facilitate the recruitment of neutrophils to the site of inflammation [110].

ROS production induces major mitochondrial DNA damages which result in defects in transcription of electron transport chain subunits and further exacerbate ROS production [111]. In addition, ROS induce mitochondrial lipid membrane deterioration which leads to the release of cytochrome C and Bax translocation to the mitochondria. These manifestations drive apoptosis in pericytes and endothelial cells in diabetes [112]. Other sources of ROS generation are NAD(P)H oxidase (NOX), cytochrome p450, and nitric oxide synthase [113]. Superoxide anion can also be generated by the uncoupled nitric oxide synthase, and by reacting with NO generates peroxynitrite, which contributes to IR [114].

One of the metabolic manifestations of hyperglycemia that enhance oxidative stress is the polyol pathway, which corresponds to the conversion of glucose to sorbitol by aldose reductase. The aldose pathway has been suggested to contribute in the pericyte loss [115]. The accumulation of AGEs also contributes to retinal damages in DR. AGEs bind with their receptors (RAGEs) in endothelial cells, pericytes, and RPE to induce NADPH-mediated oxidative stress, which in turn induces NF-*κ*B activation and cytokine formation [116].

The hexosamine pathway is another pathway that mediates the high glucose-driven oxidative stress and ensued complications observed in DR. Fructose 6-phosphate is deviated from the glycolytic pathway to be converted to glucosamine 6-phosphate and then to uridine diphosphate *N*-acetylglucosamine (UDPGlcNAc). UDPGlcNAc attaches to Ser/Thr residues leading to posttranslational modifications of proteins. The hexosamine pathway leads to the activation of plasminogen activator inhibitor-1 (PAI-1) which participates in the pathogenesis of diabetic complications [117, 118].

#### 3.2.1. Antioxidants in DR

Given the role for oxidative stress in the genesis of DR, potential effective interventions can be achieved. Under normal conditions, endogenous antioxidant systems such as superoxide dismutase (SOD), catalase, thioredoxin reductase, glutathione reductase, glutathione peroxidase, GSH, thioredoxin, and tocopherol (vitamin E) ensure the clearance and detoxification of ROS and free radicals and prevent their accumulation. In diabetic rats, these antioxidant molecules have been found to be diminished in the retina [110]. Conversely, overexpression of mitochondrial SOD can be protective against oxidative stress in the retina under diabetes conditions [119].

A number of antioxidant compounds have been shown to be protective in diabetic retinopathy animal models; however, some clinical studies have failed to demonstrate the efficacy of these antioxidants in DR [120–122]. Vitamins C and E supplementation may protect against progression of DR [123, 124]. Vitamins C and E enhance the enzymatic activities of glutathione reductase, glutathione peroxidase, and SOD and decrease pericyte dropout in diabetic rats [125]. Lipoic acid, an antioxidant which can scavenge ROS, was found to have beneficial effects in the development of DR by inhibiting capillary cell apoptosis and inhibiting oxidative damage in the retina of diabetic rats [126]. Nicanartine is a lipid-lowering compound which has antioxidant properties and was found to be effective in inhibiting pericyte loss in diabetic rats although it fails to prevent the increase in acellular capillary formation [127].

Polyphenols contained in green tea have potent antioxidant properties. Green tea supplementation increases retinal GSH levels and the enzymatic activities of catalase and SOD and decreased acellular capillaries in diabetic rats [125, 128]. Benfotiamine, a thiamine derivate (vitamin B1), has been shown to inhibit three ROS production pathways (hexosamines, PKC, and AGEs pathways) implicated in DR pathogenesis, as well as NF-*κ*B activation, and to prevent the increase in acellular capillaries in the retina of diabetic rats [129]. Some lipid-lowering drugs such as fenofibrate have been shown to be beneficial in preventing the progression of DR, given that dyslipidemia and high circulating fatty acids are associated with increased oxidative stress in the retina. Two placebo-controlled randomized trials, the Fenofibrate Intervention and Event Lowering in Diabetes and Early Treatment Diabetic Retinopathy Study (FIELD) and Action to Control Cardiovascular Risk in Diabetes (ACCORD) eye studies have shown that fenofibrate retards the progression of DR in adult patients with type 2 diabetes [130, 131]. The mechanism by which fenofibrate exerts its protective effect remains to be determined and may involve oxidative stress, apoptosis, inflammation, and BRB preservation [132], possibly by inducing anti-inflammatory effects via sirtuin 1-dependent signaling pathway inhibition of NF-*κ*B in human retinal endothelial cells [133].

### 3.3. Inflammation and DR

Inflammation has been also suggested to contribute to the development and progression of DR. Activation of Toll-like receptors by pathogen-associated molecular patterns (PAMPs) leads to the release and the nuclear translocation of NF-*κ*B which triggers the transcription of several cytokines and chemokines such as TNF*α*, IL-1, IL-6, and MCP-1. In diabetes, there is an increase in the activity of TLR2 and TLR4 which participate in microvascular complications [134]. High concentrations of glucose induce the expression of TLR2 and 4 via PKC*α* and PKC*δ*, and a knockdown of both TLR2 and TLR4 reduces high glucose-induced NF-*κ*B activation [135]. Some studies have reported that increased plasma levels of free fatty acids (FFAs) can activate TLR2 and TLR4 [136, 137]. Monocytes have been shown to respond to the presence of FFAs by increasing TLR2 and TLR4 expression resulting in increased NF-*κ*B activation [138].

Activated microglial cells are considered to be a major source of proinflammatory cytokines in damaged tissues. In diabetic rats, an increase in the number of activated microglia in the retina was associated with increased production of inflammatory cytokines, ROS and MMPs, and a concomitant loss of neuronal cells in ganglion cell layer and inner nuclear layer [139]. Likewise, several studies have shown that DR is accompanied with an augmentation of inflammatory mediators including ICAM1, VEGF, IL-6, IL-8, and MCP-1 and angiogenic factors such as angiotensin II, angiopoietin-1, angiopoietin-2, and erythropoietin (reviewed in [140]). For instance, high levels of TNF*α* have been detected in the vitreous humor and serum from DR patients [141–143]. It has been shown that TNF*α* directly contributes to BRB breakdown in DR by activating PKC-*ζ*/NF-*κ*B pathway which reduces the expression of tight junction proteins claudin-5 and ZO-1 and increases endothelial cell permeability [144]. Blocking TNF*α* actions by using the specific TNF*α* inhibitor etanercept [145] disrupts NF-*κ*B activation and inhibits BRB dysfunction.

Other than microglia, Müller cells can also contribute to the inflammatory response in DR, as these cells produce a variety of inflammatory factors [146–148]. Besides, several lines of evidence have shown that the adherence of leukocytes contributes directly to the death of endothelial cells via Fas-FasL-mediated mechanism [149]. The adhesion of leukocytes to the diabetic endothelium is mediated by the intracellular adhesion molecule-1 (ICAM-1) and CD18. Neutralization of ICAM-1 and CD18 with specific antibodies attenuates leukocyte adhesion and prevents retinal endothelial cell injury [150].

#### 3.3.1. Anti-Inflammatory Agents in DR

Despite glycemic control, blood pressure control, and lipid-lowering therapy in diabetic patients, the prevalence of DR is increasing and therapeutic approaches are limited. The development of new anti-inflammatory strategies to prevent and treat DR is being proposed. Etanercept is an FDA-approved recombinant fusion protein for the treatment of psoriasis because of its anti-TNF*α* properties [151]. Acting as a competitive inhibitor of TNF*α*, etanercept reduces leukocyte adhesion, suppresses BRB breakdown, and decreases their activation [145, 152]. Although other TNF*α* inhibitors such as pegsunercept have displayed efficacy in animal models, by reducing pericyte dropout and capillary degeneration [153, 154], etanercept failed to demonstrate efficacy in patients with diabetic macular edema refractory to anti-VEGF therapy [155].

Resveratrol (3,5,4′-trihydroxystilbene), a naturally occurring polyphenol found in grapes and red wine, is known for its antioxidant and anti-inflammatory properties. Oral resveratrol administration (5 mg/kg) has been shown to improve glucose tolerance, decrease NF-*κ*B activation, and lower TNF*α* levels in preclinical diabetic models [156]. Resveratrol also exerts its neuroprotective effects on retinal ganglion cells following intravitreal injection, improving their survival by activating sirtuin 1 [157]. Its effects have not been tested in humans with diabetes.

IL-1*β* [141, 158, 159] and the enzyme caspase-1 generating IL-1*β* are considered important targets to prevent DR [160]. Accordingly, inhibition of caspase-1 using minocycline or by blocking IL-1R1 receptor prevented diabetes-induced increase in IL-1*β* and degeneration of retinal capillaries [158]; interestingly, similar effects were seen with exogenous antioxidants [159, 161].

## 4. Age-Related Macular Degeneration (AMD)

Age-related macular degeneration (AMD) is the most common cause of vision loss in the elderly population and accounts for 8.7% of all blindness worldwide [162]. Its prevalence is increasing as a consequence of an exponential aging in the population. According to a recent systematic review and meta-analysis study [162], the projected number of people with AMD in 2020 is estimated at 196 million and approximately 288 million by 2040.

### 4.1. Pathogenesis of AMD

Two types of AMD are clinically recognized: dry AMD which is characterized by the formation of extracellular deposits called drusen, followed by RPE and photoreceptor death, and geographic atrophy (GA) and wet AMD which is characterized by choroidal neovascularization [163, 164]. Both forms of AMD result in loss of central vision. To date, laser photocoagulation and anti-VEGF therapy are the most common treatments for wet AMD [165]. However, mechanisms and, therefore, treatments for dry AMD remain largely elusive.

**Figure 3:**
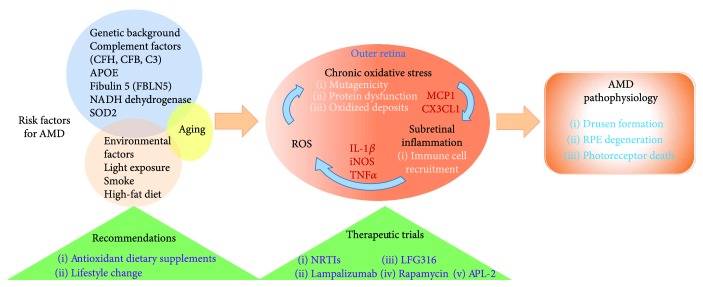
Scheme summarizing the risk factors of AMD, the link between oxidative stress and inflammatory factors involved in the pathogenesis of dry AMD and the current antioxidant/anti-inflammatory therapeutic trials and recommendations. CFH: complement factor H; CFB: complement factor B; C3: complement 3; APOE: apolipoprotein E; NADH: nicotinamide adenine dinucleotide H; SOD2: superoxide dismutase 2; ROS: reactive oxygen species; MCP1: monocyte chemotactic protein 1; CX3CL1: C-X3-C motif chemokine ligand 1; IL-1*β*: interleukine 1 beta; iNOS: inducible nitric oxide synthase; TNF*α*: tumor necrosis factor alpha; NRTI: nucleoside reverse transcriptase inhibitor; RPE: retinal pigment epithelium.

AMD is a multifactorial disorder wherein a complex interplay of genetic and environmental factors contributes to its pathogenesis (Figure 3). Multiple genes involved in lipid metabolism, complement pathway, and extracellular matrix remodeling have been found to be associated with AMD progression [6]. Furthermore, RPE senescence [166], oxidative stress [9], and immune dysfunction [167] are also involved. In the following sections, we will focus our discussion on the role of oxidative stress and inflammation in AMD.

### 4.2. Oxidative Stress and AMD

The outer retina, composed by RPE and photoreceptors, is constantly exposed to an oxidative environment, on one hand arising from the high oxygen delivery from the choroid and, on the other hand, due to constant photic bombardment. Photoreceptors are the main source of ROS in the outer retina due to light exposure and their high metabolic rate-associated oxygen consumption [168]. Outer segments of the photoreceptors which are rich in polyunsaturated fatty acid (PUFA) are sensitive to auto-oxidation and prone to oxidative stress. To maintain the balance in the proportion of oxidant species, photoreceptors have efficient antioxidant defense mechanisms such as SOD1 and SOD2 which transform superoxide (O_2_^−^) to hydrogen peroxide (H_2_O_2_) and superoxide (O_2_^−^), and glutathione peroxidase (GPx), and/or glutathione reductase (GR) and catalase which convert O_2_^−^ into H_2_O and O_2_ [9]. In addition to its enzymatic defenses, photoreceptors count on its interlink with the multifunctional RPE cells. RPE cells ensure the correct functioning of the outer retina, for instance, by maintaining the structure of the external blood-retinal barrier, secreting growth factors, absorbing excess of light, participating in the photoreceptor outer segment phagocytosis, and cycling of retinoids. RPE daily phagocytes the oxidized membranes of outer segments, contributing in this way, to decrease the oxidative stress in photoreceptors. However, the degradation products of these phagocytized outer segments generate lipofuscin in RPE cells. Due to its molecular composition, lipofuscin is photoreactive and thus increases the susceptibility of RPE to light damage with aging. In addition, phagocytosis process may also generate high level of H_2_O_2_ through NADPH oxidase activity and peroxisomal-oxidation, which exposes the RPE to the risk of oxidative stress [169, 170]. Interestingly, photoreceptors and RPE cells can modulate oxidative damage induced by oxidized cellular components with an autophagy process involving activation of p62/Nrf2 pathway [171, 172].

Many factors such as aging, environmental stress, smoke cigarette, and genetic factors are involved in the development of AMD (Figure 3). These elements may also contribute to increase the production of prooxidants and reduce antioxidant defenses. For instance, cigarette smoke contains high level of ROS [173], which has demonstrated to reduce the levels of endogenous antioxidants, such as glutathione, cysteine [174], and SOD [175].

Mutations of genes encoding proteins involved in the mitochondrial respiratory chain (NADH complex, cytochrome complex) have also been shown to lead to pathological oxidative stress increasing the risk for AMD [176]. A similar paradigm also applies to complement factor H defense protein (CFH) [177], and for polymorphisms of NADH dehydrogenase and SOD2 [178].

#### 4.2.1. Antioxidant Agents in AMD

A number of antioxidants have been suggested to confer protection of RPE and photoreceptors. This is the case for carotenoids (lutein, zeaxanthin, and mesozeazanthin) which act as scavengers of ROS [179, 180], alpha-lipoic acid which enhances GPx activity [181], curcumin which augments the expression of the cytoprotective and antioxidant enzyme heme oxygenase-1 in RPE cells, and caffeic acid phenethyl ester (CAPE) which confers photoreceptor protection against H_2_O_2_-mediated cell death by increasing the expression of heme oxygenase-1 and supressing NF-*κ*B activation [182, 183]. However, despite the promising effects of these antioxidant molecules in animal models, two large multicenter investigations, the Age-Related Eye Disease Study (AREDS and AREDS2), failed to show convincing efficacy of these types of supplementation for AMD [184, 185].

### 4.3. Inflammation and AMD

RPE cells exposed to oxidative stress can elicit inflammation [186, 187]. The accumulation of lipids, particularly in the form of drusen, is associated with a chronic inflammation. RPE cell function is crucial for retinal homeostasis, and loss of its integrity and/or function increases the risk of progression of AMD. When oxidative stress exceeds the antioxidant defense capacities, RPE cells release inflammatory chemokines such as MCP-1 and fractalkine (CX3CL1) playing a key role in microglia recruitment in subretinal space [188]. Physiologically, microglial cells are not present in the outer retina; however, their migration increases upon damage to the photoreceptors and/or RPE cells and also when insoluble debris from phagocytosis are accumulated in RPE cells and subretinal space during aging. These oxidized deposits containing advanced glycation end products (AGEs) and lipoxidation end products (ALEs) are recognized and cleared by macrophages [189, 190]. When oxidative stress is sustained, monocytes are recruited from blood circulation. These activated immune cells present in the subretinal space release IL-1*β*, iNOS, and TNF*α*, which in turn induce ROS production in RPE through NADPH oxidase activation and increase oxidative damages [191, 192].

Even more, proinflammatory cytokines secreted by infiltrating lymphocytes or macrophages exert senescence and dysfunction to RPE [193, 194]. RPE senescent cells in turn may secrete a range of pleiotropic factors that recruit inflammatory cells and exacerbate inflammation and damage to the outer retina [195]. Accordingly, accumulation of microglia in the outer retina has been associated with RPE and photoreceptor damage in AMD [196, 197]. All these evidences highlight the interwoven relationship between oxidative stress and inflammation in the development of AMD.

#### 4.3.1. Anti-Inflammatory Agents in AMD

Robust evidence suggests that modulation of inflammation could attenuate AMD progression. Rapamycin (sirolimus), an mTOR inhibitor used for its immunosuppressive effect in organ transplantation, has been proposed for AMD therapy. Sirolimus has been shown to preserve RPE and photoreceptors from cell death in a mouse model of retinal degeneration [198]. However, in a phase II clinical trial, the subconjunctival or intravitreal injections of sirolimus every 3 months for 24 months did not reduce geographic atrophy progression in the patients with AMD [199, 200].

Targeting the complement system provides another strategy to tackle inflammation and reduce AMD progression [201]. Encouraging results were observed with the humanized anti-factor D monoclonal antibody complement inhibitor, lampalizumab (Roche Inc.). These results from phase II clinical trial showed a 24% reduction in geographic atrophy after monthly intravitreal injections for 18 months [202]. Along the same lines, the C3 inhibitor APL-2 (Apellis Pharmaceuticals) and the anti-C5 monoclonal antibody LFG316 (Alcon) are currently in phase II clinical trial [203].

In the perspective of targeting inflammation to treat AMD, NLRP3-inflammasome, a key component of the innate immunity, has shown to play an important role in the development of AMD [204]. A number of studies have shown a strong association in the activation of the NLRP3-inflammasome and the development of geographic atrophy in patients with AMD [205, 206]. Interestingly, nucleoside reverse transcriptase inhibitors (NRTIs), administered for human immunodeficiency virus (HIV) patients, have proven to block P2X7-dependent NLRP3 inflammasome activation [207]; currently, clinical trials are under preparation using NRTIs for AMD patients.

## 5. Conclusion

Oxidative stress and inflammation play an important role in the development of IRs. A better understanding of the mechanisms implicated in early stages should identify new targets that allow the development of new therapeutic approaches. Along these lines, a more profound elucidation of the complex interplay of oxidative stress and inflammatory mediators is required. Although, several epidemiological and animal studies have revealed beneficial effects of antioxidants, results from clinical trials have been at best tepid, possibly because of the complexity in targeting oxidants and more importantly the absence of strategies to deal with biologically active stable product peroxidation such as isoprostanes, neuroprostanes, and isofurans [208]. Specific anti-inflammatory approaches may turn out to be more promising.
